# The Role Complexities in Advance Care Planning for End-of-Life Care—Nursing Students’ Perception of the Nursing Profession

**DOI:** 10.3390/ijerph18126574

**Published:** 2021-06-18

**Authors:** Suet Ying Ng, Eliza Lai-Yi Wong

**Affiliations:** Centre for Health Systems and Policy Research, JC School of Public Health and Primary Care, Faculty of Medicine, The Chinese University of Hong Kong, Hong Kong, China; yanng427@gmail.com

**Keywords:** advance care planning, nursing undergraduates, palliative care, end-of-life care, advance directive, ageing

## Abstract

Nurses’ perceptions of being responsible for advance care planning (ACP) vary greatly across different studies. It could, however, affect their involvement in advance care planning and patients’ quality of death. Recent studies on this topic have mostly focused on advance directives but not ACP and nurses in the ward setting. This study aimed to assess the perception of Hong Kong nursing undergraduates of the nurse’s role in advance care planning and examine its associations with knowledge, attitude, and experience. A cross-sectional 57-item survey was delivered to nursing undergraduates between June and August 2020. The chi-squared test or Fisher’s exact test were used for univariate analysis. The multiple logistic regression model was used for multivariate analysis. A total of 469 participants were assessed for eligibility; 242 of them were included in the data analysis, with a response rate of 97.6%. The majority of respondents—77.3% (95% CI: 72.0–82.6%)—perceived having a role in ACP, but large discrepancies were found between their perception of their role regarding different aspects of ACP. Participants who had a better knowledge status (*p* = 0.029) or supported the use of ACP (*p* < 0.001) were more likely to have a positive perception of their role in ACP. A negative correlation was found between the experience of life threat and positive role perception (*p* < 0.001). Through strengthening training, the role clarity of nursing undergraduates could be achieved, maximizing their cooperation with and implementation of ACP in their future nursing career. The enhancement of end-of-life education could also be undertaken to fill nursing undergraduates’ knowledge gap in this area and change their attitudes.

## 1. Conceptual Background

In end-of-life care, the nurse is regarded as one of the important healthcare professionals in the initiation and follow up on advance care planning discussions [1]. Surprisingly, a stark contrast can be observed in the percentage of nurses who regard advance care planning as part of their role across the globe. In Western countries, such as Canada, the U.S., and New Zealand, the statistics report a high level of agreement that nurses have a role in ACP, with 99% of Canadian nurses agreeing on this point [2,3,4]. In Asian countries, such as Singapore and Taiwan, the proportion of nurses perceiving ACP as part of their role is far lower: only 37% of Singapore renal nurses regard ACP as their responsibility [1,5]. There is little data on Hong Kong regarding this issue; whether a low proportion of nurses perceive ACP as their role in this Asian city remains to be seen. This discrepancy could possibly be linked to differences in nurses’ undergraduate curricula and training and the in the legislation status of advance directives in different countries. For example, the U.S. established the legislation of advance directives in all 50 states and the online module, End-of-Life Nursing Education Consortium (ELNEC), to enhance nursing undergraduates’ palliative care skills [6]. In contrast, Hong Kong does not have this legislation of advance directive and a comprehensive curriculum as the U.S. does [7].

In 2017, the American Association of Colleges of Nursing (AACN) emphasized the importance of nurses across all care settings and populations being well educated about primary palliative care, suggesting that nursing students should fulfil 17 palliative care competencies listed in the Competencies and Recommendations for Educating nursing Students (CARES) before graduation from their pre-licensure programs, promoting the adoption of the ELNEC project [8]. A total of 45,000 nursing undergraduates from 490 schools have been trained using this program since 2017, and 25% of universities have integrated this into their curricula [9]. The ELNEC undergraduate curriculum could be classified into six modules. The role of nurses throughout palliative care is discussed, from their role in the provision of care as a member of an interdisciplinary team to their role in discussions with the patient, family, and interprofessional team [10]. The flexibility and accessibility of the online program, the integration of questions, and the release of the ELNEC certificate after completion has meant that ELNEC has been incorporated into undergraduate curricula smoothly [11]. While some schools do not enroll their students into the ELNEC, their own faculty palliative care experts deliver standardized primary palliative care education, ensuring that their students satisfy the competencies required by the AACN CARES [10]. Moreover, the barriers and related strategies preventing the implementation of ELNEC projects have been identified by various studies, encouraging schools to implement the program [11]. In contrast, Hong Kong does not currently have such precise guidelines and competency requirements for end-of-life care [12,13]. According to the “Reference Guide to the Syllabus of Subjects and Requirements for the Preparation of Registered Nurse (General) in the Hong Kong Special Administrative Region” stipulated by the Nursing Council of Hong Kong, all universities are required to include end-of-life issues and share the same syllabus [12]. However, end-of-life care does not have its own section; it is possibly classified under the umbrella of gerontological care [12]. While the registered nurse syllabus only requires 40 and 20 h for legal and ethical issues and communication, respectively, these areas are not related to advance care planning [12]. The core competencies of registered nurses include only five areas, and end-of-life care is not included [13]. The current hospital authority guidelines on advance care planning only mention possible initiators, including healthcare workers, patients, and families; the description of the role delineation and specification is inadequate [14]. The legislation of advance directives and attempts to include advance directive education programs in the undergraduate curriculum have commenced in the U.S. but not in Hong Kong. However, nursing undergraduates are anticipated to play a crucial role in ACP in their future nursing career, and it is hypothesized that their medical training will be related to their perception of their role [3]. Therefore, it is important to study the perceptions of nursing undergraduates of the nurse’s role in ACP and association with other factors, as these enhance the quality of death in Hong Kong.

Currently, studies concerning advance directives and ACP are scarce, and mostly focused on advance directives across the globe [3,15,16]. Several overseas studies have revealed nurses’ perceptions of their roles and some associated factors [1,2,4,5,17]. As researchers have a greater tendency to target nurses in the ward setting as participants, nursing students are rarely recruited in studies [3]. We therefore aimed investigate the associations between the knowledge, attitude, experience, and role perception of Hong Kong nursing undergraduates and their perception of the nurse’s role in ACP in this study. The significance of this study is that it aims to enhance the preparedness of nursing undergraduates for taking a role in advance care planning in the following ways: gathering data on nursing students’ knowledge, attitude, and clinical experience; boosting nurses’ rate of initiation of advance care planning discussions; enhancing the quality of death for elderly individuals in nursing homes; and providing important information for policy makers, managers, and educators in the area of end-of-life care.

## 2. Materials and Methods

### 2.1. Design

A cross-sectional survey using a structured questionnaire was the method adopted to study nursing students’ perception of their role, knowledge, attitude, and experience related to ACP in Hong Kong. Anonymous online questionnaires were distributed through social media with a convenience sampling approach that contained a well-stratified sample, based on what year of the program the students were in. The study was approved by the Survey and Behavioral Research Ethics of The Chinese University of Hong Kong.

### 2.2. Participants

The participants were undergraduates enrolled in a government-funded pre-registration undergraduate nursing program from four universities, involving 899 students in Hong Kong based on a report from 2020 [18]. The mental health nursing program [19] and the higher diploma program in enrolled nursing [20] excluded the limited coverage of end-of-life care in their curricula. For the sample size estimation, Singapore was chosen as our reference population, as it shares many similarities with Hong Kong. They have similar rankings and ratings for the quality-of-death index [2,21]. Both are Asian settings and possess a physician-dominated model [1,22]. The confidence level anticipated was 95% with a desired relative precision of 0.05. According to the literature, the proportion nursing students perceiving that nurses played a role in ACP was 37.1% for the renal nurses working in Singapore [1]; therefore, the minimum required sample size was 183.

### 2.3. Data Collection and Measurement

Eligible participants were recruited using the convenience sampling approach through social media platforms such as Instagram and WhatsApp. Electronic written consent was obtained before the commencement of the survey; participants were required to complete a self-administered, short (less than 15 min), and anonymous questionnaire on Google Forms through the link sent to them. The instrument used was a self-administered questionnaire that consisted of 57 items grouped into five sections, including the sections demographics, perception of role, knowledge, attitude, and experience. The questionnaire was developed based on the published tool and a literature review [1,23]. An online, self-administered questionnaire was used, enabling us to reach a wider group of the target population across universities during the COVID-19 pandemic and to ensure the anonymity of the participants.

The perception of role section consisted of 9 items; 7 of them were designed in a 5-point Likert scale format (with 1 being the lowest), asking them to what extent they agreed with the sentiment that they will have a role in ACP in their future nursing career and in which aspects of it, regarding engaging patients in ACP discussion. In terms of validity, a published tool for primary care and palliative care clinicians was modified to investigate the nursing students’ perception of their role in advance care planning discussions, with the scale of 0 to 9 replacing the scale from 1 to 5, as other studies mostly measure their attitude and role perception on a scale of 1 to 5 [24]. The data were further regrouped into a yes–no format for comparison with prior research [1]. According to the literature, agree (4) and strongly agree (5) were regrouped together as role perception [2]. Respondents were asked about their expectations of who should initiate and be involved in the ACP discussion.

The knowledge section contained 16 items and participants were provided with the binary option (yes/no) to answer questions about their self-perceived knowledge. In terms of validity, most of the questions were developed based on the Singaporean study, which aimed to assess the health literacy of medical professionals in terms of end-of-life care. The mean score was calculated as an indicator of the results. In terms of reliability, the participants were asked if they had ever heard of advance care planning and were aware of the related public consultation. Participants who had never heard of ACP were instructed to skip the following session focusing on assessing their knowledge. Their score for the knowledge section was 0, avoiding inconsistent results resulting from random answers. A detailed definition was provided to them, assisting them in the completion of the subsequent section. A total of 13 questions in the knowledge section were designed in a true/false format, aiming to assess the participants’ knowledge of ACP (correct answers received 1 point; incorrect answer and “not sure” answers received 0 points). This was modified to adapt to the situation in Hong Kong. Eight was used as a cut-off point since the median knowledge score among those who had heard of ACP was 8.0/13 (61.5%).

A five-point Likert scale was used to explore the attitudes of participants toward ACP (strongly agree, agree, neutral, disagree and strongly disagree) through 19 items in this section. A total of 16 questions from two published studies were adapted to the Hong Kong setting [1,23]. Participants were also asked to rate their willingness and confidence and whether they supported the use of ACP, using a scale of 1 (lowest support) to 5 (highest support) for consistency with the previous 16 questions.

The personal information section, comprising 13 items, was further divided into the following: demographic information with 6 items, and experience related to ACP with 7 items, from experience of life threat to engagement in ACP with the binary scale (experienced/not experienced). Demographic data were collected for the identification of potential confounders, including age, gender, ethnicity, and religion.

### 2.4. Data Analysis

The IBM Statistical Package for Social Science (SPSS, IBM Corporation, Armonk, NY, USA) for Mac (Ver 25) was used for the data management and analysis. All the demographics were descriptively presented in percentages. To address the primary objective, the proportion of nurses perceiving ACP as part of their role was presented with a 95% confidence interval. For the univariate analysis, the factors associated with between-subjects perceiving and not perceiving ACP were compared using chi-square tests or Fisher’s exact test for categorical variables. Multiple logistic regression analysis was conducted to identify the independent factors associated with ACP perception. A *p*-value (*p*) < 0.05 demonstrated a significant association.

## 3. Results

### 3.1. Demographics

Four hundred and sixty-nine responses were received between June and August 2020; 221 were excluded due to not being eligible. Among the 248 participants, 6 declined to provide electronic consent and 242 were included in the study, for a final response rate of 97.6%. Open University of Hong Kong (OPENU) nursing students accounted for 33.1%, 32.6% of them were from the Chinese University of Hong Kong (CUHK), 26.4% of them were from the Hong Kong Polytechnic University (POLYU), and 7.85% of them were from the University of Hong Kong (HKU). A total of 87.6% of the students were year 3 or below, 86.8% of them were female, 32.2% of them were religious, and only 3.7% of them lived alone.

The majority of the respondents had a low awareness of ACP (47.1%); 75.6% of them were not aware that the Food and Health Bureau had launched a public consultation on AD and dying in place. Only a small proportion (13.2%) of them perceived themselves as having sufficient knowledge of ACP. Around half of them (52.9%) had never heard of ACP, thus scoring 0 for all questions in the knowledge section.

### 3.2. Role Perception of Hong Kong Nursing Undergraduates in ACP

Among the respondents, 77.3% (95% CI: 72.0–82.6%) of the Hong Kong nursing undergraduates had a positive perception of their role in ACP. The association between awareness and role perception was not further investigated, since these served as screening items. Year of study and religion were found to have a significant association with students’ perception of their role (Table 1). Students studying in their senior year were more likely to perceive nurses as having a positive role in ACP than those in their junior year (*p* = 0.02), whereas students with a religious belief were more likely to perceive nurses as having a negative role in ACP (*p* = 0.017).

The majority of the Hong Kong nursing undergraduates regarded nursers as having a proactive role in the aspects related to initiating ACP discussions with patients (72.3%), exchanging information with patients (83.5%), helping patients to communicate their ACP with their families (85.5%), and helping patients to communicate their ACP with health care professionals (89.3%). However, only around half of the respondents considered themselves as having a proactive role in being a decision coach for patients who are trying to engage in ACP (58.3%) and participating in the finalization of the ACP plan (54.1%).

### 3.3. Association between Knowledge and Role Perception in ACP

Less than half (47.1%) of the participants attempted the knowledge questions. The mean score was 3.8 out of 13 (95% CI: 3.24–4.31). The frequencies of correct responses for the 13 questions were low, ranging from 5% to 46%. The questions with the four highest frequencies of correct responses (42.1–45.9%) were those that indicated the requirement of good communication skills for ACP discussions (Q9) (45.9%), the importance of documentation in ACP (Q6) (43.4%), the patient’s life values and preferences being taken into consideration (Q2) (43.0%), and the ACP having the appropriate timing (Q7) (42.1%). The questions with the two lowest proportions of correct responses were those that referred to ACP as a legal document rather than a process (Q5, 5.0%) and which implied that the patient has to be mentally competent to carry out ACP (Q10, 9.9%).

Marginal significance was observed between the correlation of knowledge and role perception. A greater proportion of respondents with a positive role perception achieved a mean score greater than or equal to 8 compared to respondents with a negative role perception (33.2% vs. 20.0%, *p* = 0.062) (Table 2).

### 3.4. Association between Attitude and Role Perception in ACP

In general, the majority of the respondents supported the use of ACP (81.8%) and possessed a high level of willingness to join in the process of ACP (69.0%). However, a low proportion of them perceived that they had received adequate nursing education about ACP (11.6%) and few had a high level of confidence (28.5%).

Respondents with a positive role perception had a significantly higher level of willingness to join in the process of ACP compared to those with negative role perception (74.3% vs. 54.5%, *p* = 0.005). The proportion of respondents with a positive role perception who supported the use of ACP was significantly higher than those with a negative role perception (90.9% vs. 50.9%, *p* < 0.001). The proportion of respondents with a positive role perception who perceived that they had received adequate nursing education about ACP was significantly smaller than among those with a negative role perception (9.1% vs. 20.0%, *p* = 0.026)

### 3.5. Secondary Outcome: Association between Experience and Role Perception

The respondents mostly did not have experience related to ACP. The majority of them had never experienced life threats before (86.8%), did not have chronic illnesses (94.2%), had never been involved in family members’ ACP (93.0%), had never been involved in patients’ ACP (85.1%), and had never been exposed to ACP in their nursing education before (71.5%). Only around half of them had family members who had experienced life threats before (50.0%) or had family members with chronic illnesses (56.6%).

A negative relationship between experience with ACP and positive role perception was identified. The proportion of participants who had experienced life threats and had a positive role perception was significantly lower than the proportion of those with a negative role perception (9.1% vs. 27.3%, *p* < 0.001). The proportion of participants who were involved in a family member’s ACP and had a positive role perception was significantly lower than the proportion of those with a negative role perception (4.8% vs. 14.5%, *p* = 0.030).

### 3.6. Potential Factors Related to Perceived Support Role in ACP

Table 3 shows the association between positive role perception and knowledge, attitude, and experience related to ACP with adjustment for demographics. Support of the use of ACP was a significant factor with a large adjusted odds ratio of 12.259, associated with the positive self-perceived role in ACP (*p* < 0.001). Knowledge also had a positive correlation with a positive role perception of ACP, with an adjusted odds ratio of 2.687 (*p* = 0.029), while experience of life threat was found to have a negative correlation with a positive role perception of ACP, having an adjusted odds ratio of 0.169 (*p* < 0.001).

## 4. Discussion

As studies undertaken both overseas and in Hong Kong have mostly emphasized the AD and perceptions of the public and nurses in a ward setting, inconsistencies in nurses’ perceptions of their role in ACP can often be seen in overseas studies [1,2,3,4,5,16,24]. Moreover, few studies have explored the association of the role perception of ACP with knowledge, attitude, and experience [3,15,16]. This cross-sectional study showed that 77.3% of nursing undergraduates have a positive role perception in ACP. Apart from this, we also found significant associations between role perception and good knowledge, positive attitude, and experience. The results could facilitate the development of education and policy related to ACP.

### 4.1. Role Perception in ACP

This study found that a high proportion (77.3%) of Hong Kong nursing undergraduates had a positive role perception. This is an encouraging figure, implying that they generally do see themselves as advocates of ACP, similar to the nursing staff in Canada (99%) and New Zealand, unlike those in Singapore (37.1%) and Taiwan [1,2,4,5]. Role perception relating to six different aspects was further examined, as nurses think that their responsibilities include encouraging patients to talk with their families or doctors but not being decision coaches or finalizing plans [24]. This could possibly be attributed to their lack of confidence or willingness. Studies have stated that non-physician clinicians, including nurses, are more likely to have lower willingness, confidence, and involvement in being a decision coach and being involved in the finalization of an ACP plan [24].

Not only could misconceptions and uncertainties about the nurse’s role in ACP be observed in the respondents’ role perception in the six aspects, but it could also be observed in the knowledge section. In the knowledge section, most participants did not score highly in questions related to ACP implementation roles (nurses could initiate ACP, correct: 27.7%; ACP could only be carried out by a doctor, correct 27.3%). In previous studies, nurses also mentioned that as health professionals’ roles overlapped within an ACP multidisciplinary team, there was some blurring in the relationship between responsibilities [3]. The lack of role clarity regarding advance care planning in the Hong Kong government guidelines and nursing undergraduate curricula could possibly be a contextual factor. The Hong Kong government is working on the legal status of advance directives; however, as ACP is the first step of AD, the protocol for ACP could be further improved [25]. Since the hospital authority guidelines on advance care planning only mention possible initiators, including healthcare workers, patients, and families, the description of role delineation and specification is inadequate [14]. On the contrary, the AACN has been encouraging the ELNEC program, providing U.S. nursing students with a clear role in advance care planning [9]. While role clarity for nurses is required in order to achieve more effective end-of-life care decision-making [24], a clearer role definition regarding different aspects of ACP could be provided, thus achieving role clarity to a greater extent. The difference in government recognition of the nurse’s role in advance care planning could possibly be another factor. While U.S. national organizations state that nurses should be the patient’s advocate, promoting patient and family participation in decision-making, the Hong Kong Hospital Authority guidelines on advance care planning state that physicians should play the main role and nurses should assist them [14,26,27,28]. Given physicians’ capability of discussing medical issues and prognoses with patients and signing legal forms, Hong Kong nurses might also anticipate that physicians will act in this role under the influence of the physician-dominated model [23,24]. Since Asian communities tend to let physicians take the main lead, the Hong Kong nurses’ role is not being optimized [26], while the nurses’ role is being optimized in the U.S. and Canada instead [5,23].

In terms of conceptual implications, a clear role for ACP team members and core competencies of nurses in advance care planning could be established in Hong Kong. End-of-life care could be included in the core competencies of registered nurses, highlighting nurses’ role in ACP, such as that of assessor, initiator, information provider, communicator, facilitator, advocate, and manager, thus encouraging the enhancement of the undergraduate curriculum based on the ability, knowledge, skills, and attitude listed in the core-competencies [3,13,29]. Nurses are also expected to identify the patient’s process of ACP and inform the health care team, initiating the discussion as to whether a patients’ health status would require one [17]. For practical implications, the role clarity would enable staff to be more active and involved in ACP conversations, thus increasing the quality of death [30,31,32]. As a hub of the ACP team, nurses who acknowledge their roles could educate other professionals about the model of shared care and change the culture around ACP practice in their setting [17]. Given their allocation in all care settings and the acknowledgement of their being a manager in ACP, nurses could even impact on and lead the interdisciplinary team, thus eventually reaching a consensus on the patient’s situation and goals for advance care planning across all settings [17]. Studies have supported the idea that modifying ACP clinical workflows and assigning suitable duties for healthcare team members could possibly facilitate a team-based approach to ACP, maximizing patient engagement through role clarity [32]. Furthermore, research has shown that the ACP group visit model, which is a team-based approach, would be practical [32].

### 4.2. Significant Factors Related to Role Perception (Knowledge, Attitude, Experience)

A deficit in knowledge and its significant association with role perception were revealed. A positive correlation was identified between knowledge and role perception. Studies have cited that nurses feel that their lack of knowledge hinders them from discussing AD with their patients; they therefore did not perceive ACP to be part of their professional role [24]. Around half of Hong Kong nursing undergraduates have never heard of ACP, and they achieved a low overall mean score, implying that there is a lack of knowledge and awareness in this area. Hong Kong nursing undergraduates (5.99/9) had comparatively lower scores in terms of knowledge than Singaporean nurses (6.3/9) and a defined good score (6.7/9) [1]. Similar patterns of highest frequencies and lowest frequencies of correct responses could be observed in the Singapore study and this study [1]. For example, AD was often confused with ACP [1]. The low awareness of this issue could be attributed to the lack of legal status in AD, similar to Taiwan and Singapore; the awareness of health care proxies in the implementation process among nurses is not yet well organized as a result [1,5]. Apart from the undergraduate curriculum, the deficit of knowledge might also be caused by the lack of legal status of AD in Hong Kong. Studies have reported that U.S. nurses’ knowledge status surpasses that of nurses from countries where ACP legislation has not yet been launched, such as Hong Kong [33].

The knowledge gap identified here could inform policy makers, strengthening the curriculum design. Another practical implication would be that the legal status of advance directive should also be pursued in Hong Kong. An introduction to end-of-life care, such as the difference between AD and ACP aforementioned, could be incorporated into the undergraduate nursing education and training programs at work, dealing with their deficit in knowledge [34]. If end-of-life care can be included in the core competencies of Hong Kong registered nurses, the establishment of competency-based training will assist nursing undergraduates to fulfil the requirements as well [35]. This will possibly enhance their knowledge of medical legal documentation and communication skills, clarifying their misconceptions cited in this study, such as that an advance directive has to be signed prior to the initiation of advance care planning [36]. Studies have shed light on the association between nurses’ knowledge, role perception, and rate of participation in ACP. Therefore, education could reinforce the continuing application of ACP, raising the ACP utilization rate and ultimately improving the quality of death [37]. Apart from education, as organizational policies are also related to nurses’ knowledge and skills in ACP, the Hong Kong government could support the legal status of AD by raising their awareness and addressing their lack of knowledge [37]. Interdisciplinary collaboration across health and legal professions could then be encouraged to increase team members’ level of knowledge, preventing deficits in knowledge of the legal implications of ACP and AD [38]. Joint advance care planning training has been suggested to facilitate the information exchange between legal and health care professionals in continuing professional development events [39]. They would have a more comprehensive understanding of the ACP in clinic settings and the legal procedures that are required in healthcare settings, facilitating their discussion with patients and family members in ACP [39].

For attitude, supporting the use of ACP was found to possess a strong positive association with a positive role perception in the final regression model. This might be explained by the fact that the process of discussing ACP might leave nurses feeling uncomfortable, so they might be unwilling to discuss ACP [15]. They would expect doctors to play a role in implementing ACP and would not see this as their responsibility [15]. Attitude is always interrelated with exposure. The experience of a life threat was negatively correlated to a positive role perception. Inconsistencies with other studies were observed here; nurses who had cared for patients with AD or had family members that went through the completion of AD were shown to have a higher discussion rate of ACP with patients than those who did not in other studies [1]. The research cited that a nurse’s values and personal experience could possibly hinder or facilitate the implementation of ACP [33]. The results in the current study could possibly be explained by the significant association between student status and a lower degree of acceptance of AD in a local study, possibly because they are unprepared for life threats and advance directives [40].

For practical implications, the enhancement of EOL education, which was proven to be effective in changing students’ attitudes, would enable nursing undergraduates to possess a more positive attitude toward the care of patients who are dying [40]. A significant association between academic year and positive attitude toward end-of-life care was revealed; final-year students were more open and positive toward caring for a dying patient. This could be due to the continuing professional development activities and formal education, as it is supported by the statistically significant relationship between knowledge and attitude in end-of-life care [41,42]. The application of evidence-based, holistic curricula, such as the ELNEC program in the U.S., might help to create a more positive attitude through filling their knowledge gap [43]. As a result, it is suggested that the undergraduate curriculum trains nursing undergraduates to be well prepared for complicated and sensitive processes and situations in end-of-life care with positive psychological capacity [44].

### 4.3. Future Research Directions

A longitudinal study targeting recent nursing graduates with a larger sample size with interval monitoring could be carried out. A comparison with this study could then explore the variations in role perception, knowledge, attitude, and experiences between nurses and nursing undergraduates, further investigating the plausible factors, such as clinical experience, specialty, and practice settings. The results could possibly be used to develop suitable, post-continuous education for nurses, boosting the rate of initiation of advance care planning.

### 4.4. Strengths and Limitations

Regarding its limitations, this study had a relatively small sample size. Additionally, a low response rate was observed in the senior year and the University of Hong Kong. Based on the factors mentioned above, the generalizability of the Hong Kong nursing undergraduates might be limited. Due to the ongoing COVID-19 pandemic, only an online survey was conducted, thus the representativeness of the sampling might be further limited. Selection bias may have arisen due to convenience sampling, thus generating positive results for the study. Nursing undergraduates may have also been alienated by unfamiliarity with AD and recall bias might have existed; incorrect information about experience related to ACP could have been provided.

Despite the limitations, this study demonstrated several strengths. The precision was set at the 0.07 level; however, due to the variation in the proportion of nursing undergraduates who perceived themselves as having a role in advance care planning in their future nursing careers and the number of nursing students reached, the desired relative precision was lowered to 0.0527. This is also the first study to examine role perception and its association with the knowledge, attitude, and experience of Hong Kong nursing undergraduates regarding ACP. Internationally, it is also a pioneering study, examining the associations between knowledge, attitude, experience, and role perception in nursing undergraduates.

## 5. Conclusions

To conclude, we found an undefined role and lack of knowledge in nursing undergraduates; implications for future education and policies concerning ACP could be raised here. Models of shared care should be established, providing precise definitions for team relationships and responsibilities to achieve role clarity. The nurse’s role in routine ACP discussions with patients in primary care can then be optimized. By strengthening the training and role expectations, the ability of nurses to cooperate and implement ACP could also be maximized. The knowledge gaps of nursing undergraduates are identified here, and this information could be used to inform the curriculum design and further strengthen it, expanding nurses’ knowledge of their crucial role in the process of ACP. Training could be provided to fill these gaps based on which questions which had the lowest frequencies of correct responses in the survey. The experience and attitude mentioned are also important factors in ACP; the enhancement of EOL education, which was found to be effective in other studies, should be provided, enabling nursing undergraduates to have a more positive attitude.

The lack of awareness of ACP and deficit in knowledge could possibly be due to the lack of legal status in AD. Therefore, the government should play a role in enhancing nursing students’ perceptions of their role, thus supporting the legal status of AD. Public education and the dissemination of the concept of ACP is also necessary in order to raise public awareness of ACP and change the attitude of the public to remove barriers for implementing ACP.

## Figures and Tables

**Table 1:** Demographics of the Hong Kong nursing undergraduates assessed in this study.

Characteristics	Total	Role Perception		No-Role Perception		*p*-Value ^1^
	*n* = 242	*n*	(%)	*n*	(%)	
Demographics						
Age						0.697
18 or below	31 (12.8)	23	(12.3)	8	(14.5)	
19–21	167 (69.0)	128	(68.4)	39	(70.9)	
≥22	44 (18.2)	36	(19.3)	8	(14.5)	
Year of study						0.002 **
1	56 (23.1)	33	(17.6)	23	(41.8)	
2	70 (28.9)	57	(30.5)	13	(23.6)	
3	86 (35.5)	73	(39.0)	13	(23.6)	
4 or above	30 (12.4)	24	(12.8)	6	(10.9)	
University						0.596
CUHK	79 (32.6)	57	(30.5)	22	(40.0)	
HKU	19 (7.9)	15	(8.0)	4	(7.3)	
OPENU	80 (33.1)	63	(33.7)	17	(30.9)	
POLYU	64 (26.4)	52	(27.8)	12	(21.8)	
Female	210 (86.8)	162	(86.6)	48	(87.3)	0.902
Religion	78 (32.2)	53	(28.3)	25	(45.5)	0.017 *
Living alone	9 (3.7)	7	(3.7)	2	(3.6)	1.000
Role perception	187 (77.3)	-			-	-
Ever heard of ACP	114 (47.1)		90 (48.1)		24 (43.6)	
Aware of the public consultation on ACP	59 (24.4)	42	(22.5)	17	(30.9)	-
Self-perceived adequate knowledge	32 (13.2)	22	(11.8)	10	(18.2)	-

^1^ *p*-value was obtained through chi-squared test or Fisher’s exact test, depending on whether the chi-squared assumptions were violated. *: *p*-value is smaller than 0.05, **: *p*-value is smaller than 0.005.

**Table 2:** Associations between participants’ role perception and their knowledge, attitude, and experience.

Characteristics	Role Perception		No-Role Perception		*p*-Value ^1^
	*n*	(%)	*n*	(%)	
Knowledge (Mean Score ≥ 8) ^2^	62	(33.2%)	11	(20.0%)	0.062
Attitude					
High level of Willingness	139	(74.3%)	30	(54.5%)	0.005 **
High level of Confidence	52	(27.8%)	17	(30.9%)	0.654
Support the use of ACP	170	(90.9%)	28	(60.9%)	<0.001 ***
Perceived adequate nursing education about ACP	17	(9.1%)	11	(20.0%)	0.026 *
Experience					
Experienced life threat before	17	(9.1%)	15	(27.3%)	<0.001 ***
Family members experienced life threat before	95	(50.8%)	26	(47.3%)	0.645
Having chronic illnesses	10	(5.3%)	4	(7.3%)	0.528
Family members having chronic illnesses	106	(56.7%)	31	(56.4%)	0.966
Involved in family member’s ACP	9	(4.8%)	8	(14.5%)	0.030 *
Involved in patients’ ACP	26	(13.9%)	10	(18.2%)	0.433
Exposed to ACP in the nursing education	56	(29.9%)	13	(23.6%)	0.362

^1^ *p*-value was obtained through chi-squared test or Fisher’s exact test, depending on whether chi-squared assumptions were violated; ^2^ only 114 participants attempted the knowledge section. *: *p*-value smaller than 0.05, **: *p*-value smaller than 0.005.

**Table 3:** Multiple logistic model of nursing students’ role perception, knowledge, attitude, and experience.

Characteristics	Adjusted Odds Ratio	(95% CI)	*p*-Value
Demographics for adjustment			
Year 4 or above	1.211	(0.373, 3.936)	0.750
Religion	0.424	(0.200, 0.900)	0.026 *
Knowledge mean score ≥ 8	2.687	(1.105, 6.537)	0.029 *
Attitude			
Support the use of ACP	12.259	(5.138, 29.251)	<0.001 **
High level of willingness	1.433	(0.644, 3.186)	0.378
Perceived adequate nursing education about ACP	0.423	(0.151, 1.189)	0.103
*Experience*			
Experience of life threat	0.169	(0.063, 0.453)	<0.001 **
Experience of being involved in family’s ACP	0.544	(0.153, 1.935)	0.347

*: *p*-value smaller than 0.05, **: *p*-value smaller than 0.005.
